# Yawn contagion in domestic pigs (*Sus scrofa*)

**DOI:** 10.1038/s41598-020-80545-1

**Published:** 2021-01-20

**Authors:** Ivan Norscia, Elisabetta Coco, Carlo Robino, Elena Chierto, Giada Cordoni

**Affiliations:** 1 Department of Life Sciences and Systems Biology, University of Torino, Turin, Italy; 2 Department of Public Health Sciences and Pediatrics, University of Torino, Turin, Italy

**Keywords:** Zoology, Animal behaviour, Anthropology, Mimicry, Social evolution

## Abstract

Contrary to spontaneous yawning—an ancient phenomenon common to vertebrates—contagious yawning (elicited by others’ yawns) has been found only in highly social species and may reflect an emotional inter-individual connection. We investigated yawn contagion in the domestic pig, *Sus scrofa*. Owing to the complex socio-emotional and cognitive abilities of *Sus scrofa*, we posited that yawn contagion could be present in this species (Prediction 1) and influenced by individual/social factors (Prediction 2). In June-November 2018, on 104 semi-free ranging adolescent/adult pigs, 224 videos were recorded for video analysis on yawning. Kinship information was refined via genetic analyses. Statistical elaboration was conducted via GLMMs and non-parametric/randomization/cross-tabulation tests. We found yawn contagion in *Sus scrofa*, as it was more likely that pigs yawned when perceiving rather than not perceiving (yawning/control condition) others’ yawns (response peak in the first out of three minutes). Yawn contagion was more likely: (1) in response to males’ yawns; (2) as the age increased; (3) within short distance (1 m); (4) between full siblings, with no significant association between kinship and distance. The influence of kinship suggests that—as also hypothesized for *Homo sapiens*—yawn contagion might be linked with emotional communication and possibly contagion.

## Introduction

Contrary to spontaneous yawning—not elicited by others’ yawns—contagious yawning takes place when the yawn emitted by a subject (trigger) induces yawning in another subject (responder)^1^. In this respect, yawning acts as a releasing stimulus (sensu Tinbergen and Perdeck^2^). Despite some morphological variants especially described in primates (e.g. chimpanzees, *Pan troglodytes*^3^; geladas, *Theropithecus gelada*^4^; Tonkean macaques, *Macaca tonkeana* and Japanese macaques, *M. fuscata*^5^; humans, *Homo sapiens*^6,7^), spontaneous yawning is considered a plesiomorphic (ancient) trait because its basic motor pattern has been observed in a wide array of vertebrates^8^. On the other hand, contagious yawning between conspecifics has been observed so far in a relatively low number of species, which suggests that this phenomenon may have appeared more recently in vertebrate evolution^9^. In particular, the presence of yawn contagion has been found only in highly social species and seems to be linked to the type of sociality more than to the phylogenetic closeness^9^. For example, in primates yawn contagion seems not to be expressed in species with relatively low levels of affiliation or tolerance (strepsirrhines: ring-tailed lemurs, *Lemur catta*, and ruffed lemurs, *Varecia variegata*^10,11^; the cercopithecid, Japanese macaque^12^; the hominid, lowland gorilla; *Gorilla gorilla*^13,14^) whereas it is present in other (in some cases phylogenetically close) species showing higher levels of social affiliation (cercopithecids: Tonkean macaque^12^; geladas^4^; hominids: chimpanzees^15–17^; bonobo, *Pan paniscus*^13,18,19^; humans^1,6^). Yawn contagion has been also found in non-primate species characterized by high inter-individual cohesion, including mammals (wolves, *Canis lupus lupus*^20^; sheep, *Ovis aries*^21^; some groups of elephant seals, *Mirounga leonina*^22^), and one social bird species (budgerigar, *Melopsittacus undulates*^23^).

It has been hypothesized that the emergence of yawn contagion has been favored by natural selection in highly social species to enhance synchronization between individuals (spatial ranging, coordinated foraging, and sleep/wake rhythms^9^). Indeed, in both human and non-human mammals, spontaneous yawning can be linked to physiological and behavioral transitions, such as those occurring over the sleep–wake cycle or during the transitions around emotional arousal (humans, *Homo sapiens*^24^; South American sea lions, *Otaria flavescens*^25^; Verreaux’ sifaka, *Propithecus verreauxi* and ring-tailed lemurs^26^; rats, *Rattus norvegicus*^27^).

In humans and great apes individual features can influence yawn contagion. These features include: (1) age (with the yawning response increasing from the immature period to adulthood; humans^28^; chimpanzees^29^; geladas^4^); (2) sex of the trigger (females can elicit more yawns in bonobos^18^; males can elicit more yawns in chimpanzees and in humans for yawns that are heard but not seen^30,31^); (3) sex of the responder (with human females showing highest frequencies of response at least under certain conditions^32^). Moreover, social factors, such as familiarity between trigger and responder, can also affect yawn contagion (humans^33^; bonobos^18,34^; chimpanzees^17^; geladas^4^; wolves^20^). Finally, different levels in the detectability of the yawning stimulus have been hypothesized to influence the response^9,35^.

The species considered in this study is the domestic pig (*Sus scrofa*), characterized by complex cognition, psychology and sociality, with stable relationships established early in life and, at least in part, retained in adulthood^36–41^. Hence, *Sus scrofa* is a particularly suitable model to investigate yawn contagion, addressed here for the first time in this species. As the phenomenon of yawn contagion might be related to emotional communication and/or inter-individual synchronization in both humans and other animals^9^, this study on the domestic pig can be relevant to both applied research in animal welfare (especially livestock) and theoretical studies on the evolutionary convergences underlying the behavior of both humans and non-human social species.

Based on the previous framework, we formulated the following predictions.

### Prediction 1: presence of yawn contagion

Yawning in pigs can express emotional arousal and chronic stress^42^. Domestic pigs can be influenced by the emotional state of others^43,44^, and use facial expressions to communicate emotional states to others (e.g. aggressive intent) and convey information about emotional responses^39^. Hence, we predicted that domestic pigs could be influenced by others’ yawns and, more specifically, that yawn contagion could be present in *Sus scrofa*.

### Prediction 2: modulation of yawn contagion

Although in a variable way depending on the species, individual, perceptive and social factors can modulate yawn contagion^9^. We therefore predicted the levels of yawn contagion could vary depending on individual features (i.e., sex and age) and on the social and spatial proximity between pigs.

## Methods

### Study group and site

This study was conducted from June to November 2018 on semi-free ranging domestic pigs (*Sus scrofa*) at the “Ethical Farm Parva Domus”, in Cavagnolo, Turin (Italy). The animals could freely move and forage in a 13 ha area of natural grassland/woodland habitat. The study group was composed by 104 pigs (7–22 month old), 54 males and 50 females coming from 14 different litters and belonging to three mixed breeds: Parma Black, Large White, and Piedmont Black. Depending on the age, the animals had been together from 3 to 14 months. No animal showed stereotypic behavior.

The males had been all castrated via the removal of testes within their first days of life; all females were potentially reproductive. Reproductive males stayed separated from the group and therefore no reproductive male was present in the study group. Four feeding spots were available in the area where the animals were provided with food (Ciclo Unico P, SILDAMIN©) every day from 8:30 to 10:30; water was available ad libitum. The pigs could integrate their diet with roots, leaves or fruits that they could find in the environment. For individual recognition, the pigs were marked with spray Raidex© for livestock, used by the farmers to mark animals, with no manipulation because the pigs were habituated to the human presence. Different spray colors and different signs on the back and sides of the pigs were used. As a result, each individual had a unique marking, renewed every 4–7 days depending on weather (observations could not be carried out during rainy days). Due to the slaughtering suspension from June to September and the low slaughter rates (usually one individual per week), all but eight pigs were available for the whole data collection period.

### Observational data, operational definitions, and video analysis

Video recording occurred on a daily basis from around 06:00 am to 05:00 pm, spanning morning and afternoon, on 80 days in total (avoiding rainy days). Video registration occurred when animals were active and records of the observational effort/animal were kept to give priority to less observed animals so to reduce observational imbalance. HD/Full HD videos were recorded by 2 people (E.C. and a field assistant) each day via Panasonic HC-V380/V180/Sony HDR-PJ240E. Extra information, when necessary, was audio-recorded on camera, with the help of a third field assistant. As a general rule, videos lasted 10 min but always included three minutes after the last yawn. For the purpose of this study, we carried out the video-analysis on 224 videos, corresponding to 42.67 h of registration and including 493 yawning bouts.

The video-analysis started after a training phase with supervisors (IN and GC), when inter-observer reliability scores measured via Cohen’s k reached 0.81 (R function “cohen.cappa”; libraries "irr" and "psych"; R version 3.5.3; https://cran.r-project.org). The video-analyses were carried out via freeware VLC 3.0.6 and extension Jump-to-Time, allowing frame-to-frame analysis.

**Figure 1:**
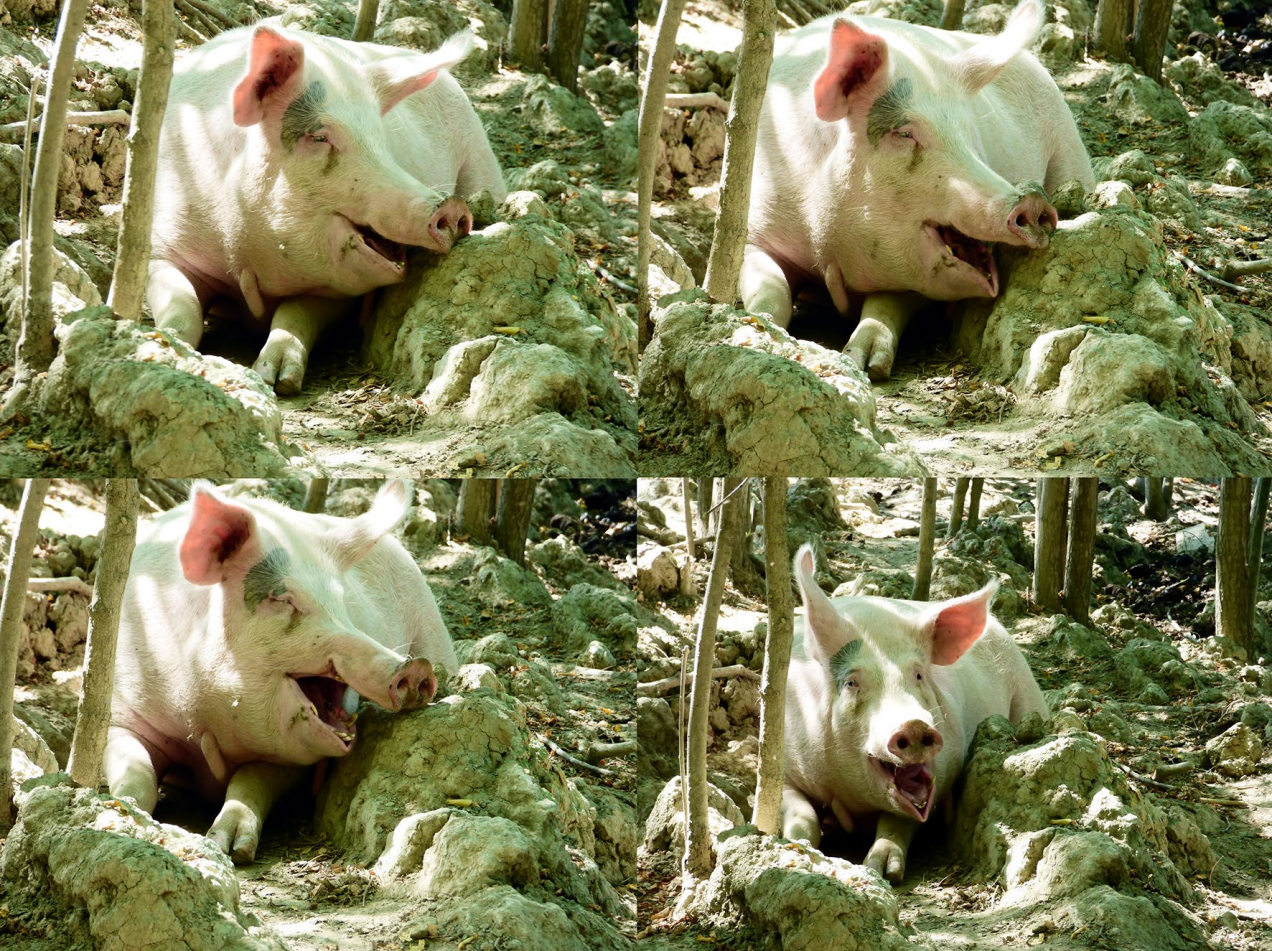
Part of a yawning sequence in *Sus scrofa* (Large White breed). Photo: Ivan Norscia (Camera: Panasonic Lumix DMC-FZ60).

Via the all occurrences sampling method^45^ (applied on videos, on the present animals), we extracted yawning bouts in absence of external unexpected events possibly generating anxiety responses (i.e. displacement activities) in the study animals. The yawning pattern involved mouth opening, with inhalation and a more rapid closing and exhalation^46^ (Fig. 1). No yawn was vocalized (via vocal folds). The possible yawning response was collected in blocks of three minutes, used in previous studies^15,18,33^, to reduce autocorrelation probability^31,47^. For each yawn, we recorded the identity of the yawner (trigger) and the identity of the animals present on videos (hereafter potential responders). For each trigger/potential responder dyad, we recorded: (1) time of the last consecutive yawn emitted by the trigger (hereafter triggering yawn); (2) number of yawns in the three minutes preceding the triggering yawn and whether they came from the trigger or not; (3) sight condition (whether the triggering yawn or other yawns emitted in the three minutes preceding the triggering yawn fell within the visual range of the potential responder or not); (4) sex, age and breed of each individual; (5) distance between trigger and potential responder (≤ 1 m, 1 < distance ≤ 10 m, > 10 m); (6) kinship (weak kinship: no parent in common; half-siblings: one parent in common; full-siblings: both parents in common); (7) minute of the yawning response in the three minutes following the triggering yawn (first/second/third). Multiple yawns from a responder in the three minutes following the triggering yawn were considered as a single response.

Owing to the wide visual range of *Sus scrofa*, a yawn emitted by an individual was considered as not visible to the other pigs only if a sight shielding, physical obstacle (e.g. fence, large tree) was present between yawner and potential responder. Doubtful cases (e.g. if it was unclear whether a pig was in the blind spot of another pig, at the back) were excluded.

Because pigs were mixed-breed, the breed was assigned based on the mother’s breed. Yawning was considered as spontaneous when a pig emitted a yawn with no previous yawns being emitted from other pigs in the preceding three minutes.

### Kinship and genetic analyses

Owing to controlled reproduction, close kinship (full siblings, maternal/paternal siblings) was known from the birth record. The different breeds, size, and marks allowed the reliable identification of the different generations. However, different mothers and fathers could be related (e.g. siblings or cousins). Therefore, to distinguish between distantly related animals (weak kinship) from more closely related ones, genetic analyses were carried out on 31 pigs, (2–3 individuals picked from different sibling generations) at the forensic genetics lab of the Department of Public Health Sciences and Pediatrics. DNA was extracted by hair bulbs (collected during the study period) via QIAmp DNA Investigator Kit (Qiagen; www.qiagen.com), provided by the farmer, following the provider’s protocol. 11 autosomic STRs were amplified via multiplex PCR Animal Type Pig PCR amplification kit (http://www.biotype.de; Biotype AG, Dresden, Germany). Genetic profile typing was obtained via capillary electrophoresis with SeqStudio system (Thermo Fisher Scientific; www.thermofisher.com). Allele frequencies and kinship index (0.08) were set on the basis of a mixed sample of domestic pigs (n = 412), consisting of commercial lines commonly used in the production process^48^. The mutation rate for all markers was set at 0.002.

For each possible dyad of pigs an unspecific kinship search was performed using Familias 3.1.5 “Blind Search” module^49^. Likelihood ratio (LR) was calculated for siblings, half-siblings, 1st cousins, and 2nd cousins relationships, scaled versus unrelated. Relationship was assigned according to the maximum LR value observed among the tested relationships.

### Statistical elaboration

The first GLMM (Model_1_) was run to test for the possible presence of yawn contagion and to verify whether yawning in the study pigs was influenced by the yawns previously emitted by group mates (n = 706). The presence/absence of yawns in the three minutes following a previous yawn was included as dependent, binomial variable (presence = 1; absence = 0). The following fixed factors were included: sight condition (factorial: 0 = previous yawning non-visible; 1 = previous yawning visible); additional yawns preceding the last triggering yawn (factorial: 0 = no previous additional yawn; 1 = one additional yawn; 2 = two additional yawns; 3 = three or more additional yawns); time slot (factorial; 1 = 6:01–9:00am; 2 = 9:01–12:00am; 3 = 12:01–15:00 pm; 4 = 15:01–17:00 pm). The identity of the potential responder was included as random factor (nominal variable).

We ran a second GLMM (Model_2_) to check what individual and social factors could influence yawn contagion. We included yawn contagion as dependent, binomial variable (1 = present; 0 = absent). As individual fixed factors we included: trigger’ and potential responder’s sex (factorial, M = male; F = female); trigger and potential’s responder age (months, numeric variable), trigger’ and potential responder’s breed (factorial variable: 1 = Piedmont Black, 2 = Parma Black, 3 = Large White). As social fixed factors we included: kinship (obtained from both genetic analyses and information on birth register: factorial; 1 = weak kinship; 2 = half siblings; 3 = full siblings); and distance (factorial; 1 = within 1 m; 2 = between 1 and 10 m; 3 = more than 10 m). The combination between trigger’ and potential responder’s identity (nominal variable) was included as random factor. For this analysis we selected the cases (n = 351) in which the triggering yawn came by one specific pig in the three min slot considered, so to single out the individual features of the trigger, and the kinship and distance between a given trigger and the potential responder. To additionally reduce the autocorrelation effect during yawn chains (subsequent yawns occurring within 3 min), only the first yawn following the last trigger’s yawn was considered as a response.

We selected the individuals with at least three yawning responses (n = 19) and calculated their yawn contagion frequencies, to check for yawn contagion distribution across the three minutes following the triggering stimulus. Owing to the non-normality of frequency distribution in the third minute (Kolmogorov–Smirnov, p = 0.001), via SPSS 20.0 we applied a non-parametric Friedman’s test for k ≥ 2 dependent samples^50^. Then, we applied the Bonferroni-Dunn post-hoc test for pairwise comparisons between minutes.

To check whether the same individual factors possibly affecting yawn contagion could also affect spontaneous yawning, a LMM (Model_3_) was run on the log_10_-transformed frequencies of spontaneous yawning emitted in each 3-min time slot by the pigs under study (n = 244; transformation applied to reach normality: Kolmogorov–Smirnov, p = 0.052). The yawner’s sex (factorial, M = male; F = female), age (months, numeric variable), and breed (factorial: 1 = Piedmont Black, 2 = Parma Black, 3 = Large White) were included as fixed factor and the yawner’s identity as random factor.

The GLMM/LMMs were fitted in R (R Core Team, 2019; version 3.5.3; https://www.R-project.org) by using the function glmer (for binary dependent variable) or lmer (for numeric dependent variable) of the R-package lme4^51^. As a first step it was verified if the full model significantly differed from the null model only including the random factors^52^. The likelihood ratio test^53^ was used to test this significance (ANOVA with argument 'Chisq'). Subsequently, by using the R-function “drop1”, the p-values for the individual predictors based on likelihood ratio tests between the full and the null model were calculated^54^. A binomial error distribution was used for binary response variables (Model_1/2_; link function: logit) whereas a Gaussian distribution was used for the normal response variable (Model_3_). A multiple contrast package (multcomp) was used to perform all pairwise comparisons between categories for significant multinomial factors with the Tukey test^55^. The Bonferroni-adjusted p-values are reported, along with estimate (Est), standard error (S.E.), and z-values.

Owing to data pseudoreplication (same individuals observed at different age), via the freeware Resampling Procedures 1.3 (package by David C. Howell) we applied a randomized bivariate correlation test (10,000 permutations) to check for the correlation between pig age and the frequency of yawning response (number of yawning responses normalized over the total number of opportunities at each age). Via SPSS 20.0, we ran the Kruskal’s lambda test to test for the association between the nominal variables distance and kinship (included in Model_2_, n = 351). P-values < 0.05 were considered as statistically significant for all tests, unless when Bonferroni correction was applied (as indicated above).

### Ethics approval

This research was purely observational and no animal manipulation was required during the study. Hence, no ethical approval was necessary according to the current regulation.

## Results

**Figure 2:**
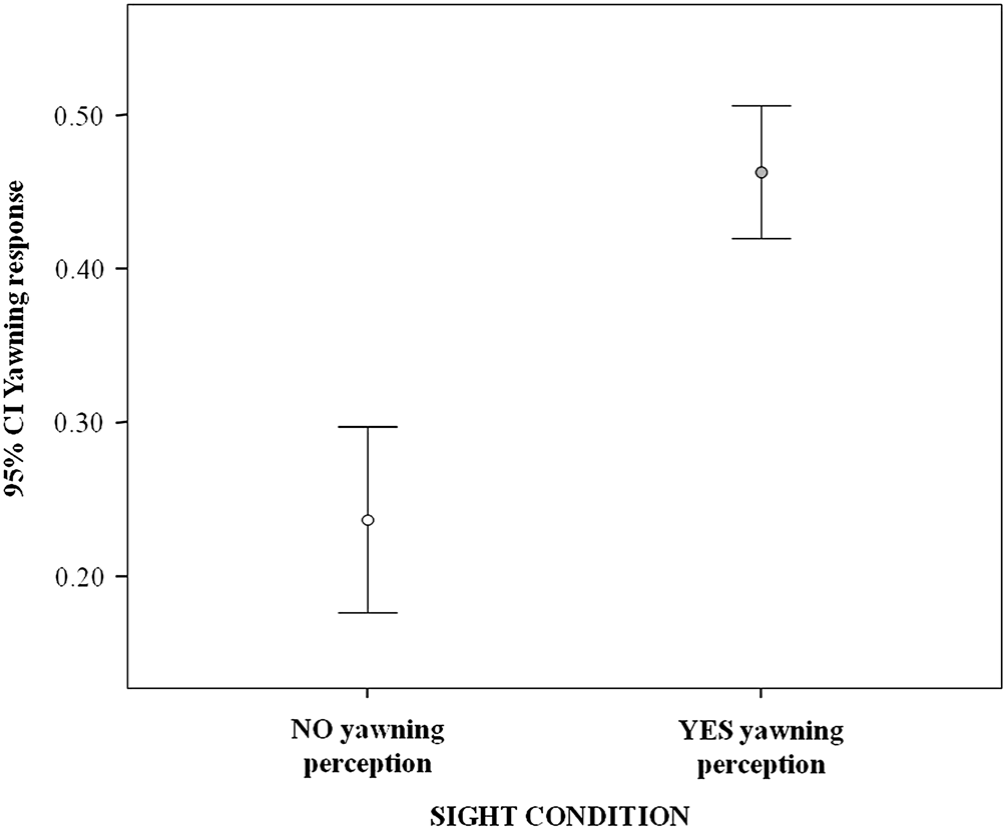
Error bars (mean, 95% confidence interval) showing the occurrence of yawning response (Y axis) as a function of the sight condition (X axis): "YES yawning perception"—the subject yawns after that another subject has yawned within its visual range (yawn condition); "NO yawning perception"—the subject yawns without perceiving a previous fellow's yawn (control condition). The variable has a significant main effect on the yawning response (results of Model_1_ included in Table 1). *CI* confidence interval.

**Figure 3:**
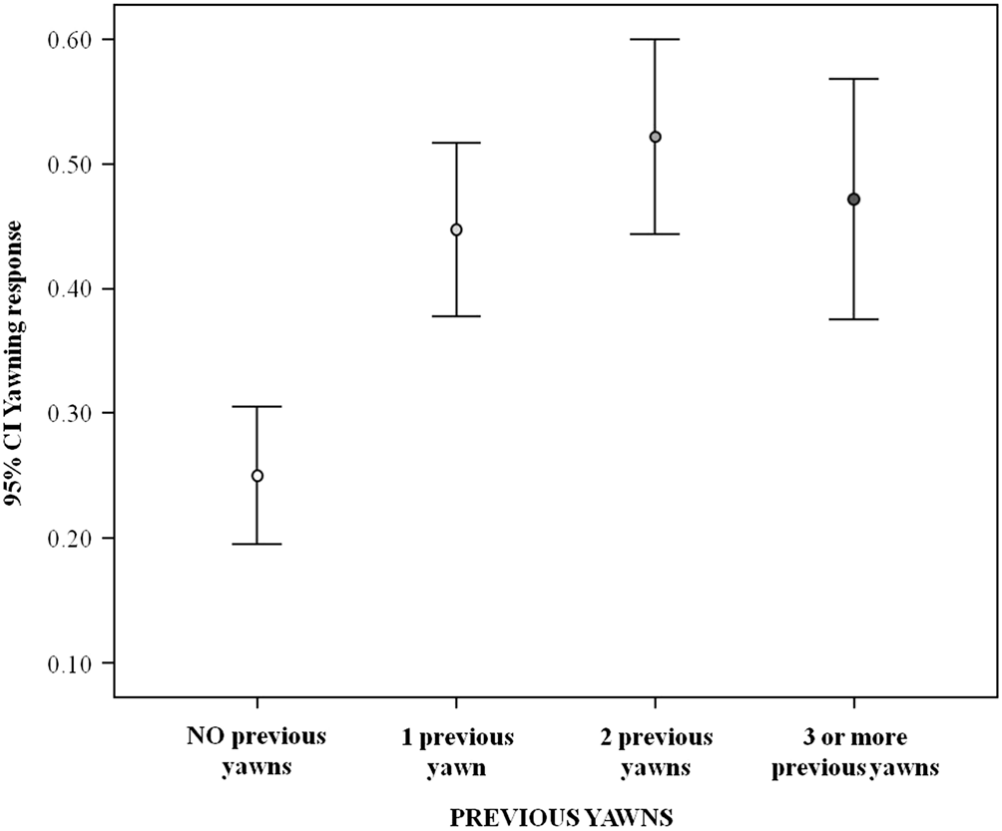
Error bars (mean, 95% confidence interval) showing the occurrence of the yawning response (Y axis) as a function of the number of additional yawns emitted by subjects other than the yawn emitted by the last trigger (X axis). The variable has a significant main effect on the yawning response (results of Model_1_ included in Table 1), with the response being highest when one or more additional yawn had been emitted compared to no additional yawn (Tukey post-hoc test with Bonferroni correction; no additional yawn versus other conditions: 0.001 < p ≤ 0.045. *CI* confidence interval.

We ran the first GLMM (Model_1_) to check whether yawning could be transferred from one pig to another and to verify what contextual factors could influence it. The dependent variable was the yawning performed by a pig within three minutes from others’ yawns (binomial: present = 1; absent = 0). We found a significant difference between the full model including all fixed factors (sight condition, daily time slot, and previously emitted yawns) and the null model, only containing the random factor (potential responder’s identity) (likelihood ratio test: χ^2^ = 72.192; df = 7; p < 0.001). Hence, we moved on with a drop1 procedure. All the tested fixed factors had a significant main effect on the dependent variable (results included in Table 1). In particular, it was significantly more likely that a pig yawned after that other pigs had yawned within their visual range rather than when the previous yawns occurred outside their visual range (Table 1; Fig. 2). Hence, yawning was contagious in the study group. Moreover, it was more likely that a pig yawned in response to others’ yawns when previous yawns had been emitted in addition to the last triggering yawn (Table 1; Fig. 3).

**Table 1:** Results of Model_1_, testing what contextual factors can modulate yawning performed by a subject within three minutes from others’ yawns and the pairwise comparisons for the multinomial, fixed factors having a significant main effect on the dependent variable.

	Estimate	*SE*	*z-*value	*P*
**Model**_**1**_**—(GLMM) Dependent variable = yawning performed by a subject within three minutes from others’ yawns (binomial: present = 1; absent = 0). Random factors = potential responder identity** **Full versus null model: χ**^**2**^** = 72.192, df = 7, p < 0.001**				
(Intercept)^a^	− 1.912	0.283	*A*	*a*
Time slot (2)^b,c^	− 0.666	0.209	− 3.192	**0.001**
Time slot (3)^b,c^	− 0.139	0.408	− 0.342	0.733
Time slot (4)^b,c^	0.248	0.285	0.871	0.384
Previous yawns (1) ^b,c^	0.817	0.236	3.456	**0.001**
Previous yawns (2)^b,c^	1.052	0.250	4.202	** < 0.001**
Previous yawns (3)^b,c^	0.865	0.283	3.060	**0.002**
Sight condition (1) ^b,c^	1.170	0.220	5.332	** < 0.001**
**Pairwise comparisons (Tukey test) for the multinomial fixed factors of Model**_**1**_** having a significant main effect on the dependent variable** **Bonferroni-adjusted p-values are reported**				
*Time slot (1 = 6:01–9:00am; 2 = 9:01–12:00am; 3 = 12:01–15:00 pm, 4 = 15:01–17:00 pm)*				
2 versus 1	− 0.666	0.209	− 3.192	**0.007**
3 versus 1	− 0.139	0.408	− 0.342	0.985
4 versus 1	0.248	0.285	0.871	0.811
3 versus 2	0.527	0.413	1.275	0.564
4 versus 2	0.914	0.295	3.096	**0.010**
4 versus 3	0.388	0.455	0.852	0.821
*Previous yawns (0 = no previous yawns; 1 = one previous yawn; 2 = two previous yawns; 3 = three or more previous yawns)*				
1 versus 0	0.817	0.236	3.456	**0.003**
2 versus 0	1.052	0.250	4.202	** < 0.001**
3 versus 0	0.865	0.283	3.060	**0.012**
2 versus 1	0.235	0.249	0.943	0.780
3 versus 1	0.048	0.279	0.143	0.998
3 versus 2	− 1.186	0.291	− 0.641	0.918

^a^Not shown as not having a meaningful interpretation.

^b^Estimate ± SE refer to the difference of the response between the reported level of this categorical predictor and the reference category of the same predictor.

^c^These predictors were dummy coded. Time slot was coded as: 1 = 6:01–9:00am; 2 = 9:01–12:00am; 3 = 12:01–15:00 pm, 4: 15:01–17:00 pm. Previous yawns were coded as: 0 = no previous additional yawns; 1 = one previous yawn; 2 = two previous yawns; 3 = three or more previous yawns. Sight condition was coded as: 0 = yawn(s) outside the visual range; 1 = yawn(s) within the visual range.

The pairwise comparison indicates that the yawning response was significantly more probable when at least one extra yawn had been emitted by other pigs (Tukey test; one additional yawn: p = 0.003; two additional yawns: p < 0.001; three or more additional yawns: p = 0.012) compared to none. No difference was found between other categories (Tukey test; p = ns; see Table 1 for full results). Finally, the occurrence of yawn contagion decreased in late morning, in the time slot 9:01–12:00 (Table 1). The pairwise comparisons revealed that the yawning response was significantly lower in the late morning (09:01–12:00am) compared to early morning (06:01–09:00am) (Tukey test; p = 0.007) and late afternoon (15:01–17:00 pm) (Tukey test; p = 0.010). No significant difference was found between the other time slots (Tukey test; p = ns; see Table 1 for full results).

**Figure 4:**
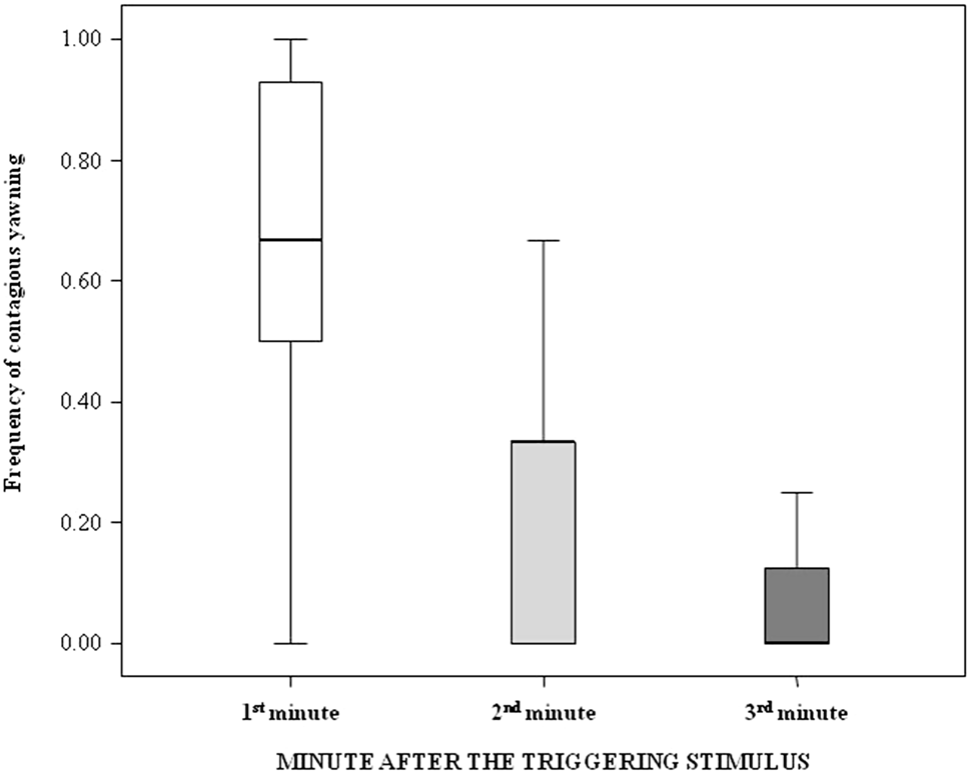
Box-plot showing the frequency of contagious yawning (Y axis) as a function of the minutes elapsing after the triggering stimulus (X axis). The difference across minutes is significant (Friedman’s test; N = 19, χ^2^ = 19.279, df = 2, p < 0.001), with yawn contagion being significantly higher in the first minute than in other minutes (pairwise comparisons via Bonferroni–Dunn post-hoc test; first versus other minutes, 0.001 ≤ p ≤ 0.045). Solid horizontal lines indicate medians, length of the boxes corresponds to inter-quartile range and thin horizontal lines indicate minimum and maximum of the observed values.

The frequencies of yawn contagion were significantly different across the three minutes from the triggering stimulus (Friedman’s test; N = 19; χ^2^ = 19.279; df = 2; p < 0.001). The pairwise comparisons revealed a significant difference between first minute and the other minutes (Bonferroni-Dunn post-hoc test; third versus first min: Q = 1.263; p < 0.001; second versus first min: Q = 0.789; p = 0.045) but not between third and second minute (Bonferroni-Dunn post-hoc test: Q = 0.474; p = 0.433). Specifically, yawn contagion was highest during the first minute (Fig. 4). Video 1 shows yawning and yawning response in two study pigs lying in contact.

As a second analysis, we carried out a GLMM (Model_2_) to verify what individual and social factors could modulate yawn contagion, whose occurrence was introduced as binomial, dependent variable (presence = 1; absence = 0). We found a significant difference between the full model, including all fixed factors (individual factors: trigger and potential responder sex, age, and breed; social factors: kinship, inter-individual distance) and the null model, only containing the random factors (trigger’ * responder’s identity) (likelihood ratio test: χ^2^ = 61.439; df = 12; p < 0.001). Hence, we moved on with a drop1 procedure.

**Figure 5:**
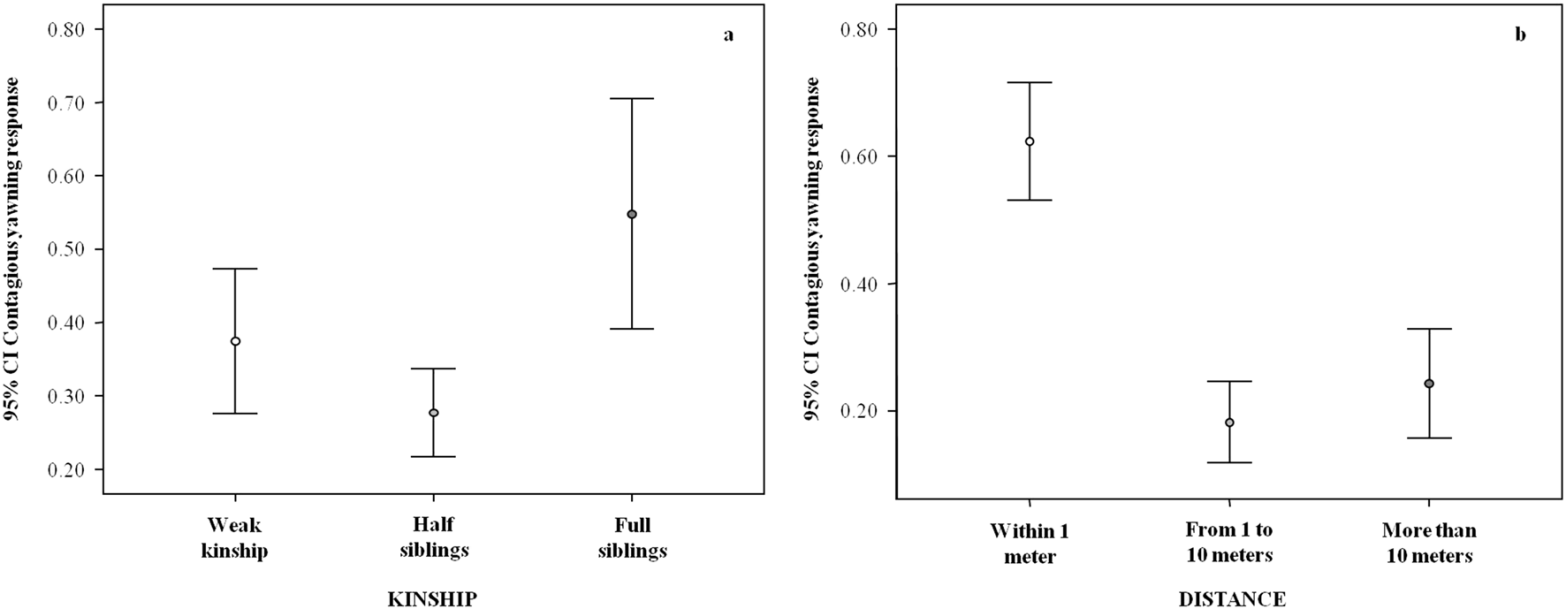
Error bars (mean, 95% confidence interval) showing the occurrence of yawn contagion (Y axis) as a function of social factors (X axis): kinship (**a**) and inter-individual distance (**b**). Both variables have a significant main effect on yawn contagion (results of Model_2_ included in Table 2), which was highest (**a**) between full siblings (Tukey post-hoc test with Bonferroni correction, full siblings versus other kinship: 0.031 ≤ p ≤ 0.033; Table 2) and (**b**) when the distance was within 1 m (Tukey post-hoc test with Bonferroni correction: within 1 m versus other distances: p < 0.05; Table 2). No significant association between the two nominal fixed factors kinship and distance was detected (Goodman and Kruskal’s lambda test: λ = 0.010, T = 0.200, p = 0.841). *CI* confidence interval.

With respect to social factors, as shown in Table 2, both kinship and inter-individual distance had a significant effect on yawn contagion. In particular, post-hoc pairwise comparisons between kinship categories revealed that yawn contagion was more likely between full siblings rather than between other categories (Tukey test; full siblings versus weak kin: p = 0.031; full versus half-siblings: p = 0.033) (Fig. 5a). No other difference was significant (see Table 2 for full results).

**Table 2:** Results of Model_2_, testing what individual and social factors can modulate yawn contagion and the pairwise comparisons for the multinomial, fixed factors having a significant main effect on the dependent variable.

	Estimate	*SE*	*z-*value	*P*
**Model**_**2**_**—(GLMM) Dependent variable = yawn contagion (binomial: presence = 1; absence = 0)** Random factors = trigger identity*responder identity **Full versus null model: χ**^**2**^** = 61.439, df = 12, p < 0.001**				
(Intercept)^a^	0.806	2.360	*A*	*a*
Trigger sex (M)^b,c^	1.867	0.792	2.357	**0.018**
Potential responder sex (M)^b,c^	0.166	0.409	0.407	0.684
Trigger age	− 0.183	0.117	− 1.563	0.118
Potential responder age	0.220	0.067	3.295	**0.001**
Trigger breed (2)	− 1.425	1.320	− 1.080	0.280
Trigger breed (3)	− 1.670	1.225	− 1.363	0.173
Responder breed (2)	− 0.329	0.852	− 0.386	0.700
Responder breed (3)	0.190	0.844	0.225	0.822
Kinship (2)^b,c^	0.449	0.800	0.561	0.574
Kinship (3)^b,c^	2.206	0.873	2.527	**0.012**
Distance (2) ^b,c^	− 2.453	0.503	− 4.872	** < 0.001**
Distance (3)	− 2.736	0.601	− 4.550	** < 0.001**
**Pairwise comparisons (Tukey test) for the multinomial fixed factors of Model**_**2**_** having a significant main effect on the dependent variable** **Bonferroni-adjusted p-values are reported**				
*Kinship (1 = weak kinship; 2 = half-siblings; 3 = full-siblings)*				
2 versus 1	0.449	0.800	0.561	0.840
3 versus 1	2.206	0.873	2.527	**0.031**
3 versus 2	1.757	0.702	2.502	**0.033**
*Distance (1 = within 1 m; 2 = from 1 to 10 m; 3 = more than 10 m)*				
2 versus 1	− 2.453	0.503	− 4.872	** < 0.001**
3 versus 1	− 2.736	0.602	− 4.550	** < 0.001**
3 versus 2	− 0.284	0.557	− 0.510	0.865

^a^Not shown as not having a meaningful interpretation.

^b^Estimate ± SE refer to the difference of the response between the reported level of this categorical predictor and the reference category of the same predictor.

^c^These predictors were dummy coded. For both trigger and potential responder: M = male, F = female; Breed: 1 = Piedmont Black; 2 = Parma Black; 3 = Large White. Kinship: 1 = weak kinship; 2 = half-siblings; 3 = full-siblings; Distance: 1 = within 1 m; 2 = from 1 to 10 m; 3 = more than 10 m.

Post-hoc pairwise comparisons on inter-individual distance indicate that yawn contagion is significantly higher when individuals are within one meter from each other, compared to further distances (Tukey test; one meter versus within 10 m: p < 0.001; one meter versus more than 10 m: p < 0.001) (Fig. 5b). No difference was found between other distances (see Table 2 for full results). Goodman and Kruskal’s lambda test revealed no significant association between the two nominal fixed factors kinship and distance (λ = 0.010; T = 0.200; p = 0.841).

**Figure 6:**
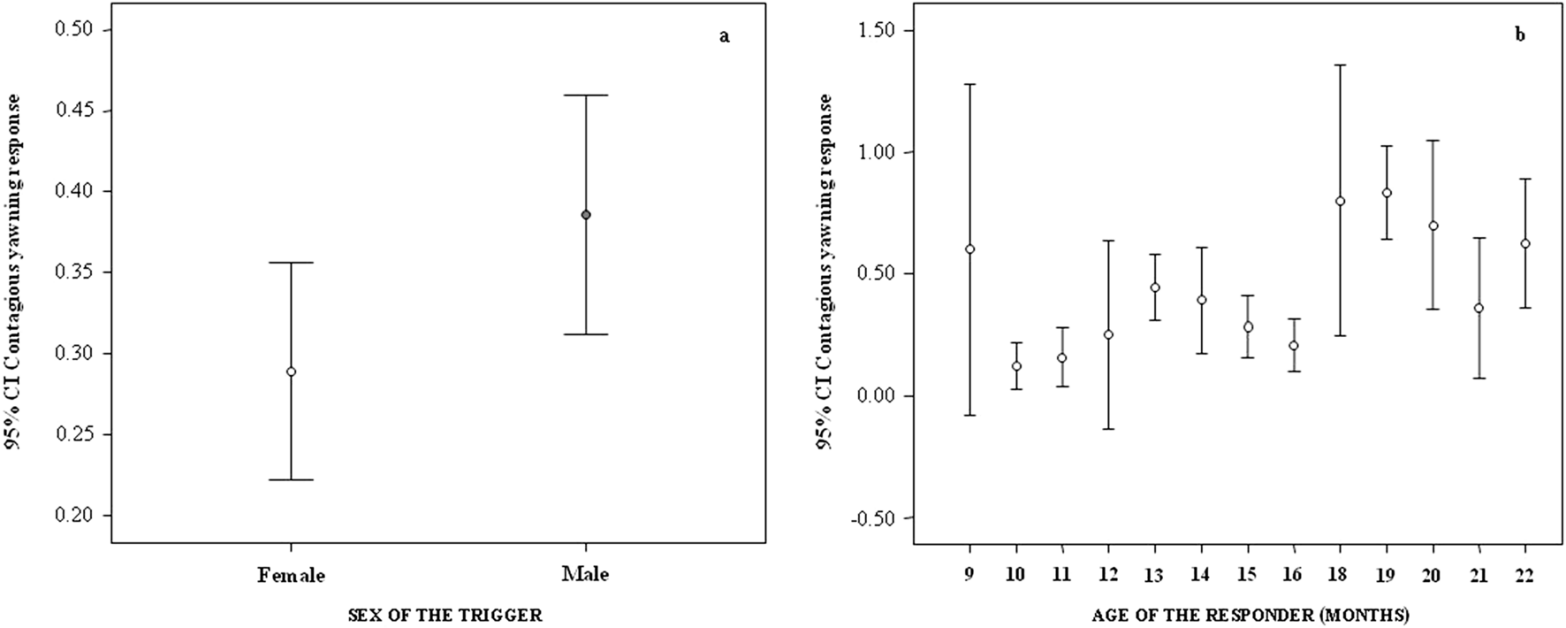
Error bars (mean, 95% confidence interval) showing the occurrence of yawn contagion (Y axis) as a function of individual factors (X axis): sex of the trigger (**a**) and age of the responder (**b**). Both variables have a significant main effect on yawn contagion (results of Model_2_ included in Table 2) with (**a**) yawn contagion being highest when the trigger was a male and (**b**) linearly increasing with age (bivariate correlation via randomization: n = 13, r = 0.600, p = 0.031). *CI* confidence interval.

As concerns the individual factors, the trigger and responder breed had no effect on yawn contagion (Table 2). Instead the sex of the trigger had a significant effect on yawn contagion, which was significantly more likely if the yawning stimulus was emitted by a male rather than by a female (Table 2; Fig. 6a). Moreover the responder’s age had a significant main effect on yawn contagion (Table 2). In particular, yawn contagion increased as age increased (bivariate correlation via randomization: n = 13; r = 0.600; p = 0.031; Fig. 6b).

Finally, a LMM (Model_3_) was run as a further control, to check whether spontaneous yawning was affected by the same individual variables affecting yawn contagion. Individual spontaneous yawning frequencies in the different time slots were included as dependent, numeric variable. We found no significant difference between the full model (including all fixed factors: yawner’s sex, age, and breed) and the null model including only the identity of the yawner (likelihood ratio test: χ^2^ = 4.924; df = 4; p = 0.295). Therefore, we proceeded no further, as the considered fixed factors had no significant effect on spontaneous yawning (sex: F = 1.302; p = 0.257; age: F = 3.419; p = 0.067; breed: F = 0.098; p = 0.907).

## Discussion

Our results show that yawn contagion is present in the domestic pig. As a matter of fact, it was significantly more likely that the pigs yawned after that at least one yawn had been emitted by another pig within their visual range (sight condition) rather than when a physical obstacle prevented the potential responder to see the yawning stimulus (control condition; Prediction 1 confirmed; Table 1; Fig. 2). Moreover, similarly as in other mammalian species^21,34^, pig yawn contagion was most likely in the first minute from the emission of the triggering stimulus (Fig. 4).

Pigs—as other highly social mammal species showing yawn contagion (for a review^9^)—can naturally form groups and engage in stable social relationships^40,41^. Immature individuals start to fine tune their relationship with others via play, first with littermates and later also with individuals from other litters^37–39^. As it has been hypothesized for other species^9^, it is possible that yawning has been co-opted during evolution to become a signal of behavioral/physiological change that other group mates could catch and replicate for reciprocal synchronization, important for social life.

Our results converge in indicating that the detection of the yawning stimulus has a crucial role in determining the yawning response, which is significantly enhanced if more than one triggering yawn is emitted (Table 1, Fig. 3) and when the potential responder is in proximity to the trigger (≤ 1 m, Table 2; Fig. 5b). This finding can be explained by the fact that pigs (which also rely on olfaction, touch, and hearing to orient themselves in the environment) possess a broad visual range (owing to lateralized eyes) but poor visual acuity^56–60^. Considering that no yawn was vocalized, pigs could only rely on vision to spot mouth opening. In this respect, any element increasing stimulus detectability (such as more yawning stimuli or short distances) could enhance the yawning response.

In humans, yawn contagion significantly fluctuates during the day, with peacks in the morning and in the evening, possibly associated with the wake-sleep cycle^61^. In chimpanzees, weak variations in yawn contagion have been observed^16^. In pigs, we observed that yawn contagion was lowest in late morning (Table 1) probably because the pigs were fed in this time slot and spent most of the time feeding and, then, sleeping. This difference is likely to disappear in the wild, where animals are not provisioned with food. In our study, the variation in the occurrence of yawn contagion was not significant across the other time slots. Thus, we cannot state that there is an appreciable variation of the phenomenon across the whole day, as expected for a species that—although preferring crepuscular and night activity—can be active over the 24h^56^.

Our findings indicate that the breed did not influence contagious yawning (Table 2). To our knowledge there is no study addressing the possible effect of breeds on yawn contagion in mammals. With the present data, it is not possible to determine whether our results mean that the yawning response is not affected by breed or whether the absence of a significant effect is due to the fact that the breeds of our study individuals were mixed.

Taken together, our results also show that while contagious yawning was significantly influenced by the sex of the trigger (with males eliciting more yawns than females; Table 2, Fig. 6a) and by the age of the responder (with yawn contagion increasing as age increased, Table 2, Fig. 6b), spontaneous yawning was not. Hence, yawn contagion probably was not enhanced by trigger males or responder’s age as a result of generally higher yawning levels in males or older pigs. Inter-sexual biases in the power of eliciting a yawning response have been found in bonobos, where yawning in group mates can be induced most frequently by females^18^, and in humans (for vocalized yawns perceived only by hearing^31^) and chimpanzees^30^ where males as triggers are particularly effective in triggering others’ yawns. In humans, for vocalized yawns that are heard but not seen, it is possible that men’s vocalizations are better heard than women’s in natural settings, often characterized by background noises^31^. In chimpanzees and bonobos, the sex bias has been related to the dominance or social relevance of the triggering subjects, considering that in chimpanzees males are dominant^30^ whereas in bonobos females acquire leadership by forming coalitions^18^. Even though castrated males can still fight for dominance^62^, it is unlikely that dominance provides a possible explanation for enhanced yawning response in *Sus scrofa*. As a matter of fact, at the adaptive level the dominant status of males may be not as much relevant for yawn contagion in the light of the species biology. Under natural conditions, in both feral pigs and wild boars females with offspring form matrilineal units and join together in stable groups of variable size whereas solitary adult males live isolated and only temporarily join female groups^40,63,64^. This social structure can explain why adult females and not males form a linear hierarchy, with older sows being dominant over younger sows^65–68^. In this perspective, a possible explanation for adult males being best triggers might be the necessity of other pigs to synchronise with reproductive males when they temporarily join the social groups composed by sows and offspring. However, further investigation on this issue is necessary to verify this possibility or formulate other hypotheses.

With respect to age, in pigs we observed a significant increase of yawn contagion as age increased (Table 2; Fig. 6b). In some primate species, yawn contagion is present in adults and absent in infants (humans^28^; chimpanzees^30^; geladas^4^). In humans, yawn contagion seems also to decline with age in adults^69^ although no conclusive results are available (e.g. see^32^). Our study subjects had passed the age in which pigs (if reproductive) reach sexual maturity (around 6 months), but it should be considered that in pigs body development continues up to 18 months, with the period between 6 and 18 months sometimes referred to as adolescence^70^. Pigs and humans are thought to share similar brain growth and development patterns, with pig brain considered closer to the human brain than other non-human animal models in terms of size, structure, and composition^71–73^. Ryan et al.^74^ found that longitudinal effects in magnetic resonance spectroscopy (MRS) measurements in adolescent female pigs (from pubescent to sexually mature) were similar to those reported in adolescent humans. Moreover, the domestic pigs possess complex cognitive and affective skills, including object discrimination, spatial learning, understanding human cues, emotional sharing, and possible elements of perspective taking^43,75,76^ (for review^36^). Hence, as it has been hypothesized for other species, the increased rates of yawn contagion in pigs with age might also suggest ongoing maturation of socio-cognitive skills and/or neural networks involved in the elaboration of social cues, developmental changes in action-understanding or identification of others’ affective state^4,29^.

Finally, in the pigs under study yawn contagion was highest between full siblings than in more weakly related individuals (Table 2; Fig. 5a). Among our study animals, 88% of full siblings came from the same litter and therefore they had spent more time than others in close association. Even though kinship and social bonding are distinct aspects^41,77^, the kinship bias observed in pigs might have a similar effect on yawn contagion as the relationship quality bias observed in other species, in which yawn contagion was highest between strongly bonded individuals (humans^33^; bonobos^18,34^; chimpanzees^17^; geladas^4^; wolves^20^). Even if the issue is still under debate^78^, it has been hypothesized that the familiarity and/or kinship bias observed in yawn contagion might reflect a form of emotional contagion, a basic building block of empathy (for review^9^). Indeed, domestic pigs show emotional contagion potentials and the ability to convey social information via facial expressions^39,43^.

As explained above, in our study we found that the spatial proximity between pigs also enhanced yawn contagion (Fig. 5b), probably by increasing the detection probability of the yawning stimulus. Social closeness and tight kinship often go in tandem with spatial closeness, with physical proximity being frequently used as a measure of social and emotional engagement in social animals^79^. We found that spatial distance and kinship between trigger and potential responder were not significantly associated with one another. Consistently, a recent study^41^ found that social proximity and relatedness were not correlated in pigs. Thus, the yawn contagion bias observed in pigs, might be linked to inter-individual relatedness, and not just proximity. This possibility can have interesting repercussions for animal welfare studies investigating emotional connection between individuals. However, no conclusion can be drawn at this stage of knowledge and further investigations, experimentally disentangling distance, kinship and social bond, are necessary to determine whether yawn contagion may be driven by familiarity per se or not.

## Supplementary Information

Supplementary Video 1.

## Data Availability

The datasets generated during and/or analysed during the current study are available from the corresponding author on reasonable request.
